# Endoplasmic reticulum stress in diseases

**DOI:** 10.1002/mco2.701

**Published:** 2024-08-26

**Authors:** Yingying Liu, Chunling Xu, Renjun Gu, Ruiqin Han, Ziyu Li, Xianrong Xu

**Affiliations:** ^1^ Department of Aviation Clinical Medicine, Air Force Medical Center PLA Beijing China; ^2^ School of Pharmaceutical Sciences Tsinghua University Beijing China; ^3^ School of Chinese Medicine Nanjing University of Chinese Medicine Nanjing China; ^4^ Department of Gastroenterology and Hepatology Jinling Hospital Medical School of Nanjing University Nanjing China; ^5^ State Key Laboratory of Medical Molecular Biology Department of Biochemistry and Molecular Biology Institute of Basic Medical Sciences Chinese Academy of Medical Sciences and Peking Union Medical College Beijing China; ^6^ School of Acupuncture and Tuina School of Regimen and Rehabilitation Nanjing University of Chinese Medicine Nanjing China

**Keywords:** diseases, endoplasmic reticulum stress (ER stress), therapeutic strategies, unfolded protein response (UPR)

## Abstract

The endoplasmic reticulum (ER) is a key organelle in eukaryotic cells, responsible for a wide range of vital functions, including the modification, folding, and trafficking of proteins, as well as the biosynthesis of lipids and the maintenance of intracellular calcium homeostasis. A variety of factors can disrupt the function of the ER, leading to the aggregation of unfolded and misfolded proteins within its confines and the induction of ER stress. A conserved cascade of signaling events known as the unfolded protein response (UPR) has evolved to relieve the burden within the ER and restore ER homeostasis. However, these processes can culminate in cell death while ER stress is sustained over an extended period and at elevated levels. This review summarizes the potential role of ER stress and the UPR in determining cell fate and function in various diseases, including cardiovascular diseases, neurodegenerative diseases, metabolic diseases, autoimmune diseases, fibrotic diseases, viral infections, and cancer. It also puts forward that the manipulation of this intricate signaling pathway may represent a novel target for drug discovery and innovative therapeutic strategies in the context of human diseases.

## Graphical abstract

The endoplasmic reticulum (ER) serves as a vital organelle in eukaryotic cells, performing essential roles such as protein folding, modification, and trafficking, lipid biosynthesis, and calcium regulation. Various conditions may disrupt ER function, potentially leading to the accumulation of unfolded and misfolded proteins, and triggering ER stress. This prompts the unfolded protein response (UPR), a conserved signaling cascade, to mitigate the stress by regulating gene expression and protein synthesis, aiming to restore ER equilibrium. However, prolonged or severe ER stress can lead to cell death. As our understanding of the molecular mechanisms linking ER stress to human diseases improves, ER stress and UPR activation are increasingly recognized as significant contributors to various diseases. The balancing of ER stress and the UPR is crucial for the pathogenesis of diseases, such as cardiovascular diseases, neurodegenerative diseases, metabolic diseases, autoimmune diseases, fibrotic diseases, viral infections, and cancer. It also considers how manipulation of this complex signaling pathway may provide a new target for drug discovery and innovative therapeutic strategies in human disease.

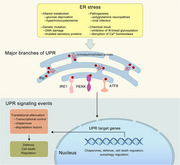

## 1 INTRODUCTION

The endoplasmic reticulum (ER) is the largest organelle in the cell, and its structure and function are intricate and multifaceted. The structure of the ER was first elucidated in 1945 when it was observed to exhibit a lace‐like appearance in electron microscope. The term “endoplasmic reticulum” was first coined by Porter and Kallman in 1952 to describe the vesicular structures observed in the pericytoplasmic region of the nucleus.^1^ The ER is divided into two types according to the morphology, structure, and function. One is found in high‐density components and is characterized by a spherical and vesicular structure, namely the rough ER. The other is located in low‐density components and is tubular in shape, designated as the smooth ER. The ER lumen is a distinctive environment, exhibiting specific attributes. It serves to maintain a calcium‐rich environment by acting as a reservoir for the high level of calcium ions in the cell, facilitated by active transport via sarco/ER Ca^2+^‐ATPase.^2^ The ER lumen has a lot of calcium‐dependent molecular chaperones, including glucose‐regulated protein 94 kDa (GRP94), glucose‐regulated protein 78 kDa (GRP78, also known as BiP), and calreticulin, which play a crucial role in the stabilizing protein folding throughout the secretory pathway. The ER lumen is an oxidative environment beneficial for the formation of disulfide bonds catalyzed by protein disulfide isomerase (PDI).^3^ Moreover, some posttranslational modifications happen in the ER, including glycosylation and lipidation, thereby reinforcing the significance of this organelle in the maturation of proteins. In addition, the ER is implicated in the synthesis of lipids, including glycerophospholipids, cholesterol, and ceramides. Moreover, the ER serves as a signaling hub, capable of releasing sequestered calcium ions that are sensitive to second messengers such as inositol 1,4,5‐triphosphate (InsP3),^4^ as well as protein kinases,^5^ and other modulators. This diverse functionality serves to illustrate the central role of the ER as both a biosynthetic powerhouse and a critical signaling organelle within the cell. Furthermore, the ER engages in extensive interactions with neighboring organelles including mitochondria, phagosomes, endosomes, lysosomes, and the plasma membrane.^6^ This complex network of interactions highlights the integrated nature of cellular function and the central role of the ER in orchestrating diverse physiological processes within the cell.

It would appear that cells operate at the limits of their secretory capacity, irrespective of the size of their ER. As a result, the workload on the ER protein‐folding apparatus often exceeds its capacity. The build‐up of unfolded and misfolded proteins in the ER results in a toxic state, which is known as ER stress.^7^ Upon detecting the accumulation of unfolded and misfolded proteins, the sensors on the ER membrane promote the cell to initiate the unfolded protein response (UPR). This process regulates ER stress and may induce related signal transduction to reduce the accumulation of unfolded and misfolded proteins by increasing molecular chaperones, inhibiting protein translation, and accelerating the degradation of unfolded and misfolded proteins. However, in the event of prolonged stress, the ER also initiates apoptotic signaling pathways, including the apoptotic transcription factor the C/EBP homologous protein (CHOP, also known as DDIT3),^8^ the activation of c‐Jun N‐terminal kinase (JNK), and caspase‐12 cleavage.^9^ Furthermore, B‐cell lymphoma group 2 (BCL‐2) proteins, which are expressed in the ER and mitochondria, are also involved in the regulation of cell death caused by ER stress.^10^ Therefore, it is currently considered that the ER is an important organelle in determining the fate of the cell. As the components of the UPR and their mechanism are elucidated, genetic investigations demonstrate the significant involvement of UPR signaling in numerous disease processes, including cardiovascular diseases (CVDs), neurodegenerative diseases, metabolic diseases, autoimmune diseases, fibrotic diseases, viral infections, and cancer.^11^, ^12^, ^13^, ^14^ Given the critical role of ER stress in the pathogenesis of multiple diseases, therapeutic strategies focusing on ER stress, particularly the maladaptive UPR, are emerging as promising avenues for disease intervention. Several small molecules have been identified that are capable of targeting the UPR and the abnormal proteins. For example, compounds that reduce ER stress by mimicking the function of certain proteins have been identified.^15^ Other methods for the reduction of ER stress include gene therapy, exercise, and calorie restriction.^16^, ^17^

This review presents a summary of the latest advances on how cells respond to stress and how this relates to the UPR signaling pathways based on human and animal models. Subsequently, the review focuses on key signaling pathways and elucidates the pathophysiology of ER stress in various diseases, including CVDs, neurodegenerative diseases, metabolic diseases, autoimmune diseases, fibrotic diseases, viral infections, and cancer. In light of the above, we review the current therapeutic approaches in preclinical development and clinical trials that target key molecules of UPR signaling pathways. Finally, we present a discussion of the remaining challenges and future research perspectives in targeting UPR regulators to treat different diseases. This review aims to provide a comprehensive account of the underlying mechanisms and signaling pathways involved in various diseases. This novel perspective on diseases has the potential to extend current knowledge of disease regulation and facilitate opportunities for drug discovery and the development of new therapeutic strategies in related disorders.

## 2 ER STRESS AND THE UPR

As a multifunctional organelle, the ER relies on a diverse array of proteins, unique physical structures, and coordination mechanisms to adapt to changes in the cellular environment. The functionality of specialized secretory cells, including pancreatic acinar and islet β‐cells, plasma cells, and rapidly proliferating malignant cells, depends on an ER environment that is conducive to efficient protein folding and quality control. A surveillance mechanism is in place to make sure that only correctly folded proteins progress through the secretory pathway. Furthermore, given the significant variations in protein synthesis and secretion rates across different conditions and cell types, a heterogeneous range of nutrient levels and energy resources is essential to meet the demands of protein folding. However, a variety of disturbances can disrupt the function of protein folding of ER, resulting in the accumulation of unfolded and misfolded proteins within its confines, thereby inducing ER stress. These disturbances encompass a range of factors, including but not limited to ischemia, hypoxia, nutrient deprivation, oxidative stress, overexpression of normal or misfolded proteins, mutation of secreted proteins, disturbance in calcium homeostasis, and viral infection.^18^ It has been demonstrated that glucose deprivation induces ER stress by impeding N‐linked protein glycosylation. Moreover, abnormalities in lipid and calcium metabolism constitute a significant contributory factor to hepatic ER stress in the context of obesity.^19^ The available evidence indicates the possibility of a correlation between high‐fat diets and ER stress in the hypothalamus.^20^ Oxidized low‐density lipoproteins have been found to induce ER stress in vascular cells.^21^ Homocysteine has been shown to induce ER stress and to reduce the secretion and expression of extracellular superoxide dismutase (EC‐SOD) in vascular smooth muscle cells. This results in increased oxidative stress in the vascular wall.^22^ Some pathogenic processes have been demonstrated to be associated with ER stress. Amyloid‐β, a protein inclusion body, that is characteristic of many chronic neurodegenerative diseases, has been found to indirectly contribute to the buildup of unfolded proteins within the ER.^23^ Furthermore, disturbances in cellular redox regulation, such as oxidants, reducing agents, or hypoxia, can lead to protein misfolding and unfolding.^24^, ^25^ Abnormalities in ER calcium regulation have been demonstrated to result in protein unfolding. consequently, the disruption of essential cellular processes that are critical for cell survival and apoptosis.^26^ In addition, several agents have been demonstrated to induce ER stress including oxidants, dithiothreitol (a thiol‐reducing reagent), thapsigargin (an inhibitor of sarco‐ER Ca^2+^‐ATPases), brefeldin A (an inhibitor of ER‐Golgi transport), and tunicamycin (an inhibitor of N‐linked glycosylation). Moreover, gene mutations of transmembrane or secretory proteins can also result in the activation of ER stress.^27^ Physiological states such as pancreatic β cell development and B lymphocyte differentiation into plasma cells, and pathological states such as ischemia/reperfusion injury, cardiac hypertrophy, and nutrient deprivation can also induce ER stress. Viral infection can result in ER stress through specific mechanisms. Evidence suggests that SARS‐CoV‐2 ORF3a may induce ERphagy through the HMGB1–BECN1 signaling pathway to cause ER stress and inflammation during SARS‐CoV‐2 infection.^28^ Collectively, stressed ER causes proteins to build up incorrectly. However, cells can react to defend against it before it is too late.

**FIGURE 1:**
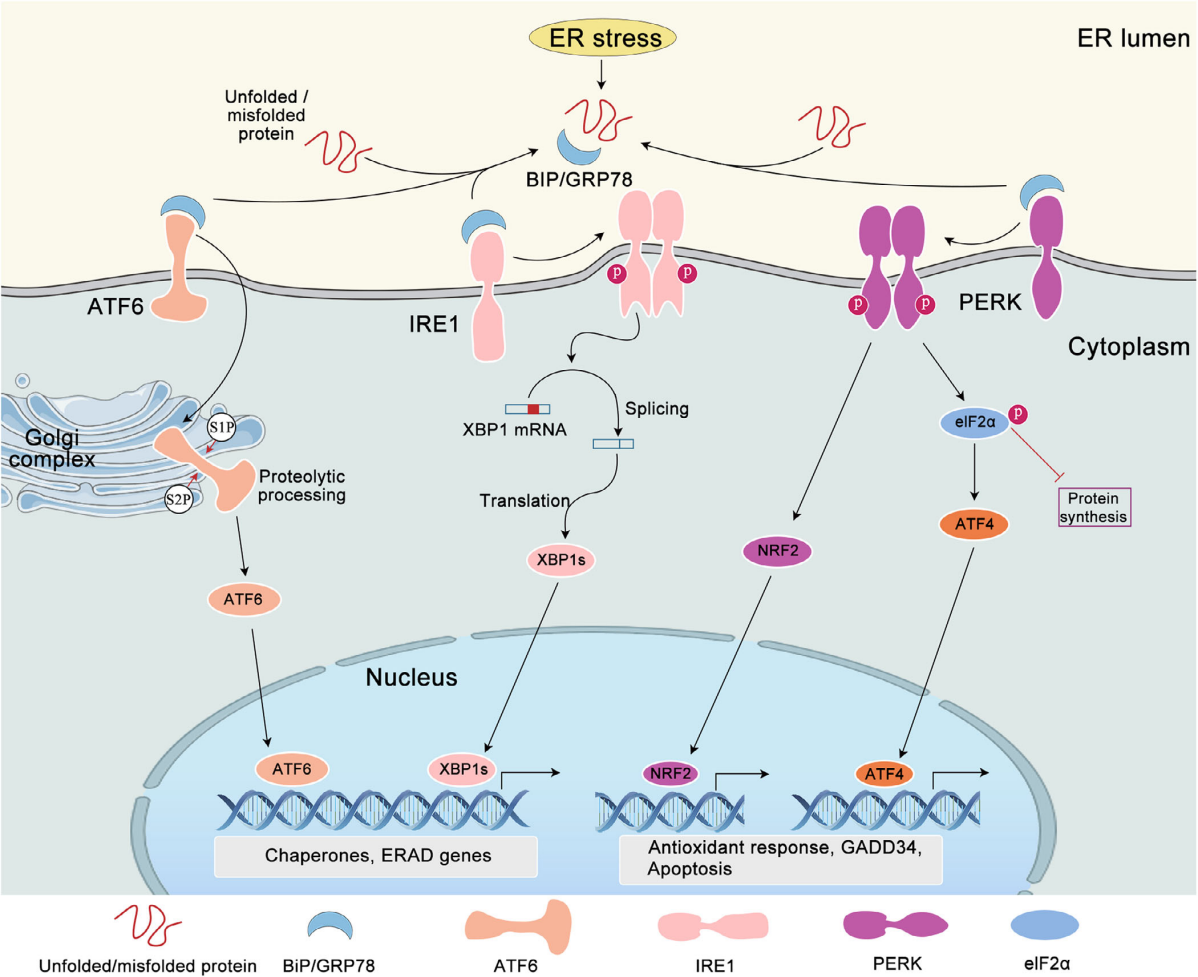
Unfolded protein signaling pathways. Many factors can cause the accumulation of unfolded and misfolded proteins in the ER to activate ER stress in the cell. The mechanism of the UPR induction is thought to culminate at the point of BiP interaction with the ER‐resident stress sensors IRE1, ATF6, and PERK. Unfolded proteins titrate BiP away from interactions with these sensors, allowing the UPR response to be activated. The activation of these three molecules has the net effect of reducing the load of proteins entering the ER by inhibiting translation and increasing the efflux of nascent proteins by either facilitating their folding through enhanced chaperone production or promoting their degradation, leading to the defense, death, or regulation of the cell.

The UPR helps the cell to survive by getting rid of excess client proteins and restoring the balance of ER. There are three main sensors of UPR pathways in metazoans: inositol requirement kinase 1 (IRE1), protein kinase‐like ER kinase (PERK), and transcription factor activating transcription factor 6 (ATF6) (Figure 1). IRE1 and PERK are similar type I transmembrane proteins with kinase domains. ATF6α is a type II transmembrane protein with a cytosolic cAMP‐responsive element (CRE)‐binding protein/activating transcription factor (CREB/ATF‐1). The N‐terminal of these three UPR sensors is in the ER membrane, while the C‐terminal is in the cytoplasm, thus establishing a connection between the ER lumen and the cytoplasm. In unstressed cells, both luminal domains of IRE1 and PERK form stable complexes with the ER chaperone BiP. Disrupting protein folding causes BiP to leave the lumen of IRE1 and PERK, but this can be reversed,^29^ which then allows IRE1 and PERK homodimerization, thereby initiating the UPR.^30^ Recent studies have demonstrated that BiP undergoes a transition from its role as a molecular chaperone to that of an ER stress sensor when the nucleotide‐binding domain of BiP interacts with the luminal domains of IRE1α and PERK, thereby facilitating further activation of the UPR.^31^ Furthermore, a direct sensing mechanism has been put forth, wherein PERK and IRE1α are postulated to directly bind to misfolded proteins, thereby leading to their oligomerization and conformational activation.^32^ Conversely, the aggregation of misfolded proteins within the ER lumen results in ATF6 relocation to the Golgi complex. Subsequently, the protein is cleaved by the proteases S1P and S2P, which release a free cytoplasmic domain that then acts as an active transcription factor.^33^ Subsequent ATF6 is activated by the dissociation of BiP from ATF6.^34^ Although the adaptive UPR serves as the primary defense mechanism against ER stress to maintain homeostasis of ER, prolonged or excessive UPR activation may result in a maladaptive state, ultimately leading to irreversible cellular injury and death.

### 2.1 IRE1 signaling pathway

IRE1 proteins, which include IRE1α and IRE1β, possess both an ER luminal sensor domain and a cytosolic endoribonuclease (RNase) domain.^35^ The precise mechanism through which IRE1 detects ER stress remains to be fully elucidated. Normally, BiP binds to the lumen of IRE1. In the context of ER stress, BiP binds to unfolded/misfolded proteins, reducing its affinity for IRE1, which enables IRE1 to undergo self‐association.^31^ IRE1 has two main functions: kinase activity and endoribonuclease activity. The kinase activity enables trans‐autophosphorylation, which activates the endoribonuclease activity. This then catalyzes unconventional splicing of XBP1 mRNA,^36^ removing an inhibitory 26‐nucleotide intron from the XBP1 transcript. Subsequently, the mRNA is religated by RNA terminal phosphorylase B, resulting in the production of the spliced XBP1 (XBP1s) mRNA, which can be translated into the transcription factor XBP1s. In turn, this induces the expression of numerous UPR genes,^37^, ^38^ which encode proteins to assist in coping with stress. They are involved in the transport of proteins from the ER to the cytosol, protein folding, protein quality control, and ER‐associated protein degradation (ERAD).^39^ Furthermore, the endonuclease activity of IRE1 can also mediate mRNA decay, thereby alleviating the burden of protein folding on the ER, a process termed IRE1‐dependent decay (RIDD).^40^ Although both IRE1α and IRE1β isoforms respond to ER stress, IRE1α displays greater XBP1 splicing activity compared with IRE1β. Nevertheless, IRE1β can act as a dominant‐negative regulator of IRE1α, effectively inhibiting XBP1 splicing despite its comparatively weaker activity in this regard.^35^

### 2.2 PERK signaling pathway

The luminal domain of PERK exhibits homology to that of IRE1, thereby indicating that they share a comparable mechanism of activation.^41^, ^42^ The dimerization of PERK on the ER membrane results in autophosphorylation and activation of its kinase domain. PERK induces phosphorylation of Ser51 of the α‐subunit of eukaryotic initiation factor 2 (eIF2α‐P), thereby inhibiting mRNA transcription and decreasing the buildup of unfolded protein in the ER. eIF2α‐P recruits to a phosphorylated insert loop that is shared with other eIF2α kinases.^43^ Some kinases that sense stress also act on eIF2α, which then has downstream effects known as the integrated stress response (ISR).^44^ However, the translation of some mRNAs is more efficient, such as the transcription factor ATF4 mRNA. The ATF4 target genes include ER molecular chaperones GRP78 and GRP79, those related to glutathione biosynthesis and antioxidant function, and those related to amino acid metabolism and transport.^44^ The ATF4 target genes can boost the level of molecular chaperones, get cellular homeostasis back on track, and help the folding and degradation of ER proteins. Additionally, ATF4 activates CHOP, which then forms heterodimers with ATF4 to upregulate genes involved in the UPR, mRNA translation, and autophagy.^45^ Once ER protein folding homeostasis is restored to its normal state, ATF4 and CHOP induce the transcription of growth arrest and DNA‐damage‐inducible protein 34 (GADD34). This protein complex with G‐actin and protein phosphatase (PP1) directs the dephosphorylation of eIF2α to terminate the ISR and restore global mRNA translation.^46^ This is vital for cells to survive when they are damaged. PPP1R15B is a paralog of PPP1R15A that serves as a buffer for low levels of eIF2α phosphorylation.^47^

### 2.3 ATF6 signaling pathway

The ATF6 transcription factor is a member of the basic leucine zipper (bZIP) transcription factor family. The ATF6α and ATF6β proteins are type II transmembrane proteins, which contain a bZIP motif in their cytosolic domain and localized at the ER in unstressed cells. The ATF6 protein contains an ER‐targeting hydrophobic sequence that facilitates its anchoring to the ER membrane. In the event of ER stress, ATF6 is released from its interaction with BiP and enters the Golgi apparatus via the coat protein II complex. Subsequently, the protein is cleaved by site 1 protease (S1P) and site 2 protease (S2P) to remove the luminal and transmembrane domains. This process results in the generation of a cytosolic fragment with transcription factor activity, which is designated as ATF6f.^33^ Subsequently, the activated ATF6 enters the nucleus, where it regulates the expression of genes encoding BiP, XBP1s, and ERAD components.^48^ The identified or postulated target genes of ATF6α include BiP, ER degradation‐enhancing α‐mannosidase‐like protein 1 (EDEM1), and PDI, which can increase the activity of ER chaperones and degradation of misfolded proteins. The transcriptional activity of ATF6β is less pronounced than that of ATF6α,^49^ indicating a potential role in dominant‐negative regulation. The antagonistic potential of ATF6β has been demonstrated through knockdown in cell culture and gene ablation in mice.^50^

## 3 CELL FATE DECISION UNDER UPR

The mammalian UPR has evolved into a dynamic and flexible network of signaling events that respond to various inputs over a wide range of basal metabolic states. In the context of ER stress, the activation of the UPR serves to alleviate the burden of unfolded proteins through the implementation of prosurvival mechanisms, thereby facilitating the adaptation and recovery of cells to stress. However, the UPR also initiates pathways that result in cell death. While some of the molecules and mechanisms involved have been identified, the full picture of how they interact to induce cell death remains elusive.

### 3.1 Adaptive UPR

**FIGURE 2:**
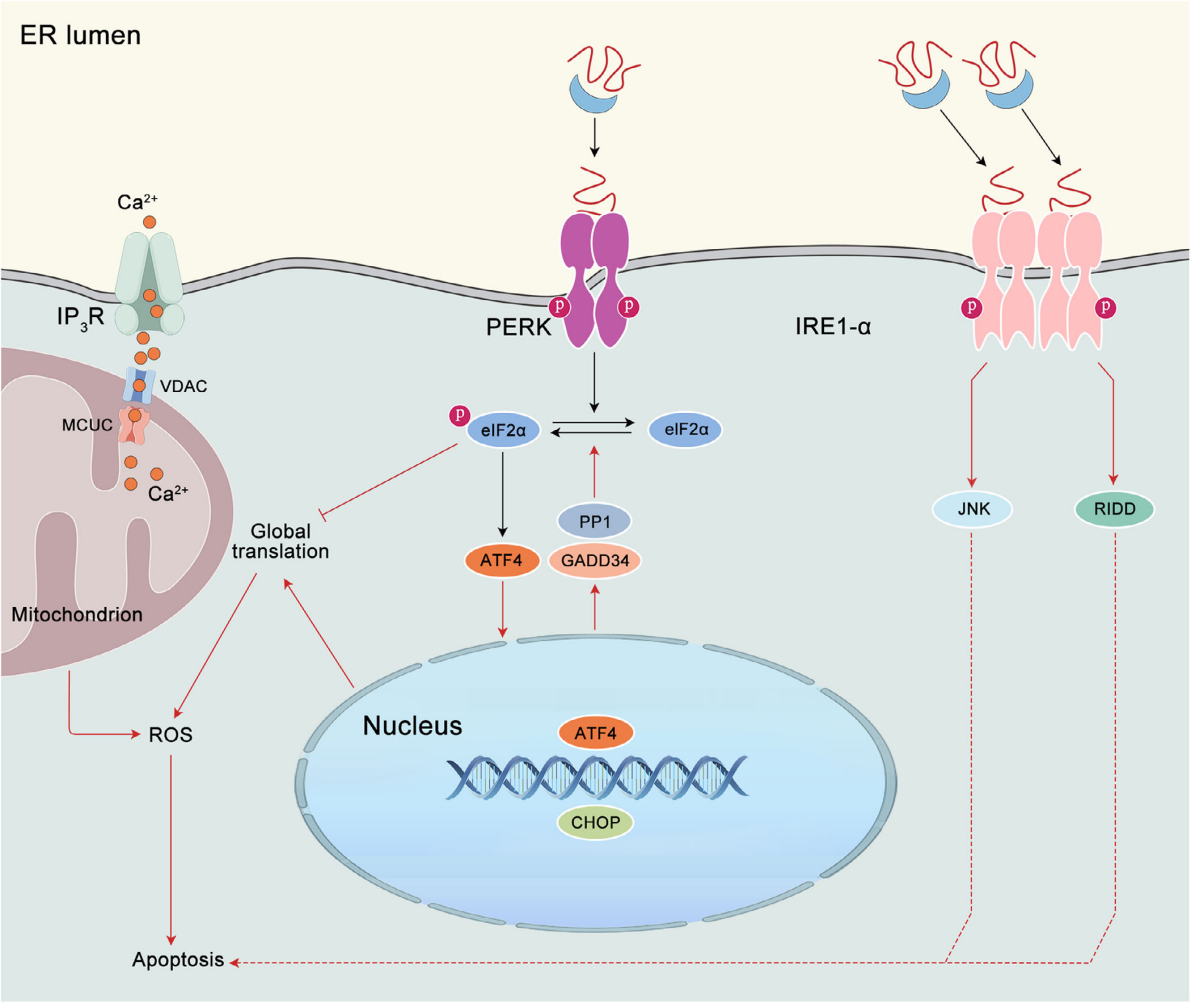
The adaptive UPR. The maladaptive UPR is triggered by persistent activation of the PERK pathway, which occurs due to prolonged and severe ER stress, ultimately resulting in apoptosis (solid red lines). The involvement of IRE1α‐induced JNK and regulated RIDD in ER stress‐induced apoptosis remains uncertain (dashed red lines).

The UPR serves to alleviate the burden of unfolded proteins within the cell, thereby facilitating survival when the ER is subjected to stress. The principal objective of the UPR is to reinstate cellular equilibrium and restore normal ER functionality (Figure 2). The UPR is important for normal cell function, especially in cells that make a lot of proteins, like pancreatic β cells, plasma cells, and hepatocytes.^51^ Two waves of cell responses have been seen in vertebrate cells under ER stress. When cells are stressed, PERK is activated, which stops general protein production. At the same time, RIDD breaks down mRNA that makes proteins in the ER.^52^, ^53^ Furthermore, ER stress also induces autophagy,^54^ a cellular degradation pathway designed to clear damaged ER and abnormal protein aggregates via the lysosomal pathway. Ultimately, quality control and degradation stop some proteins from entering the ER during translation. This allows cells to fix themselves. A second wave of adaptive UPR mechanism mainly involves the activation of genes that help the ER to fold proteins and those involved in ERAD. Each UPR sensor activates a transcription factor and upregulates specific target genes of UPR. IRE1α processes the mRNA of XBP1 to create a functional transcription factor called XBP1s, which then moves to the nucleus and controls the expression of its target genes.^55^, ^56^ The target genes encode proteins involved in cell processes like ERAD, protein folding, and protein entry into the ER. XBP1s also control the production of phospholipids, which are needed to expand the ER membrane during periods of ER stress. The ATF6 protein is cleaved in the Golgi apparatus, resulting in the release of a cytosolic fragment called ATF6f in the context of ER stress. This ATF6f controls the expression of genes such as XBP1 and those involved in ERAD.^57^ In conclusion, PERK‐mediated phosphorylation of eIF2α leads to the translation of mRNA encoding the transcription factor ATF4, which modulates the expression of genes associated with protein synthesis and folding, amino acid metabolism, and redox control.^58^ This branch of the UPR has been demonstrated to regulate a number of microRNAs, which may play a role in the inhibition of protein translation or synthesis.^59^ To sum up, these mechanisms prevent the influx of proteins into the ER, thereby enabling adaptive mechanisms to reinstate ER homeostasis.

### 3.2 Chronic ER stress and apoptosis

If the compensatory responses initiated by the UPR prove inadequate, particularly in instances of prolonged or excessive ER stress resulting from protein misfolding, cellular demise ensues, typically through the process of apoptosis (Figure 2). The intricate regulatory process that governs apoptosis in instances of irreparable ER stress remains inadequately elucidated. It is important to acknowledge that the mechanism of cell death initiated under conditions of chronic ER stress is intricate and encompasses both caspase‐dependent apoptosis and caspase‐independent necrosis. One potential mechanism regulating apoptosis is CHOP induced by PERK and ATF4.^60^, ^61^ It has been demonstrated by research that CHOP facilitates the upregulation of proapoptotic genes, including BIM, TRB3, DR5, and PUMA. Concurrently, it suppresses the expression of BCL‐2, thereby triggering apoptosis in response to ER stress.^58^ The Ddit3 deletion from cells has been observed to mitigate protein aggregation within the ER, thereby diminishing both oxidative stress and apoptosis induced by ER stress.^62^ Furthermore, ER stress induces proteotoxicity and oxidative stress, which ultimately results in cell death via a CHOP‐dependent mechanism. PERK has two distinct functions in the apoptotic process. First, it maintains the levels of CHOP. Second, its tethering function facilitates the transfer of reactive oxygen species (ROS) signals between the ER and mitochondria.^63^ In cells lacking CHOP and GADD34, the accumulation of high‐molecular‐weight protein complexes in the stressed ER is reduced in comparison with wild‐type cells. Furthermore, mice lacking GADD34‐directed eIF2α dephosphorylation demonstrate resistance to renal toxicity induced by tunicamycin. Consequently, the ER in CHOP deletion cells subjected to stress exhibits a comparatively reduced oxidizing environment. Promotion hypo‐oxidizing state in the ER, either through pharmacological or genetic approaches, has been observed to attenuate the formation of abnormal protein complexes in the stressed ER, conferring protection against the detrimental effects of ER stress. Thus, CHOP deletion serves to protect cells from ER stress by reducing the load of ER client proteins and altering the redox conditions within the organelle.^64^ Notwithstanding the rigorous regulation of calcium release from the ER via calcium‐release channels ryanodine receptors (RyR) and inositol 1,4,5‐triphosphate receptors (InsP3R), a plethora of stressors has been observed to elicit a depletion of ER calcium levels and subsequent cytosolic calcium overload. This increase in cytoplasmic calcium levels can induce apoptosis via aberrant activation of calpain or the cytoplasmic phosphatase calcineurin,^65^, ^66^ alongside the activation of ER‐resident caspases or induction of mitochondrial dysfunction.^23^ Alternatively, IRE1α has been found to induce apoptosis through the activation of mitogen‐activated protein kinases (MAPKs), which then engage the BCL‐2 family members, and/or RIDD.^52^ The precise mechanism by which ER stress is an adaptive response to a cytotoxic one remains incompletely elucidated. Given the disparate and opposing outcomes that ER stress can yield, it is imperative to comprehend the mechanisms by which UPR sensors modulate their signaling outputs to decipher the determination of divergent cellular fate.

## 4 ER STRESS IN DISEASES

**FIGURE 3:**
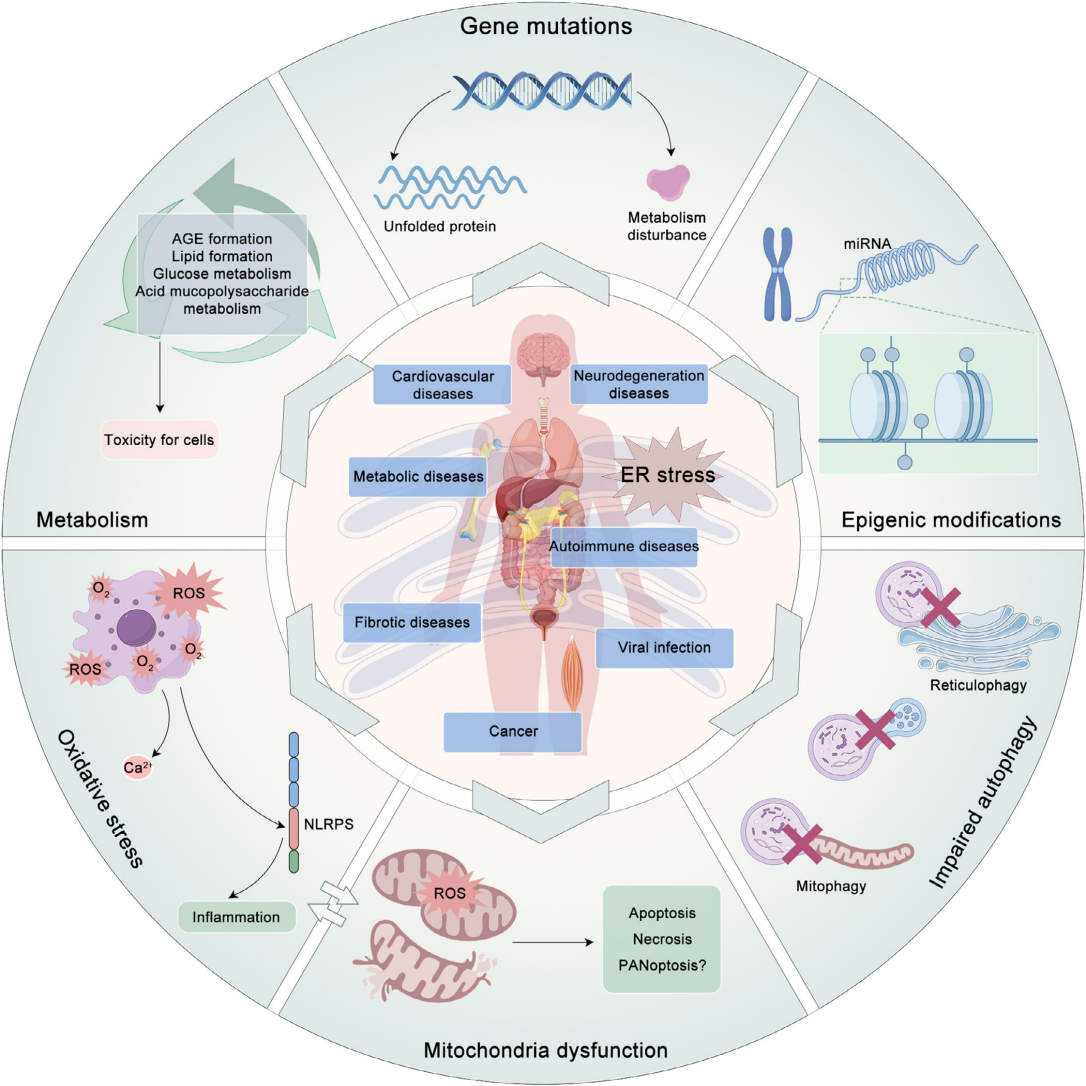
An overview of the regulatory mechanism of ER stress in diseases. ER stress plays a crucial role in many diseases, including cardiovascular diseases, neurodegeneration diseases, metabolic diseases, autoimmune diseases, fibrotic diseases, viral infections, and cancer. The specific mechanisms by which ER stress contributes to disease include various factors such as genetic mutations, epigenetic modifications, impaired autophagy, oxidative stress, mitochondrial dysfunction, and alterations in metabolism. The figure is created according to the elements in Ref. 67

Over the past decade, the evidence indicates that ER stress is a key factor in a wide range of pathological processes, including CVDs, neurodegenerative diseases, metabolic diseases, autoimmune diseases, fibrotic diseases, viral infections, and cancer (Figure 3). Although these diseases may appear to be heterogeneous, a common underlying theme can be discerned. The presence of intracellular and/or extracellular conditions impedes the protein folding process, resulting in the accumulation of misfolded proteins within the ER. Given that ER stress and the associated UPR can precipitate cell death, it is unsurprising that conditions leading to an increase in protein misfolding or a decrease in the ability of cells to handle these proteins within the ER can result in cellular dysfunction and disease. This concept is supported by the observed correlation between inherited mutations in the UPR pathway and the development of rare forms of diabetes and other diseases in humans. The following sections present detailed discussions of selected examples to elucidate the potential role of ER stress in pathogenesis, the UPR signaling pathways proposed to be involved, and potential therapeutic avenues.

### 4.1 Cardiovascular diseases

CVDs represent a significant global health burden, with a range of established risk factors, including obesity, elevated blood pressure, high cholesterol, diabetes, tobacco use, and physical inactivity. The early detection, modification of lifestyle habits, and implementation of medical interventions can facilitate the effective prevention and management of CVDs. The pathophysiological factors occurring in CVDs, including hypoxia, metabolic rearrangements, myocardial ischemia, inflammation, diabetes mellitus, hypertension, and heart failure, present significant challenge to ER homeostasis. This causes the build‐up of misfolded proteins and disrupts the function of ER, which then triggers ER stress.^68^, ^69^ The homeostasis of ER is intricately linked to normal cardiovascular function. Imbalances in ER homeostasis can initiate the onset of CVDs, which, in turn, exacerbates ER stress, thus perpetuating a detrimental cycle. ER stress can be observed to play a dual role in biological systems. In some cases, it may be adaptive, triggering the UPR that aids in cellular adaptation. However, in other instances, it may be maladaptive, resulting in dysregulated secretory pathways and contributing to the development of cardiovascular pathology. ER stress is both a causative factor and a consequence of several CVDs, including hypertension, ischemic heart disease, cardiac hypertrophy, heart failure, stroke, and diverse cardiomyopathies. The canonical and noncanonical signaling pathways of ER stress in cardiovascular pathology are context‐dependent, with potential protective or promotional effects contingent on the cellular milieu and disease progression.^11^ The latest research findings indicate that traditional ER stress sensors, such as IRE1α, may also serve as noncanonical functions. It is unanticipated that IRE1α is a structural determinant of mitochondria‐associated membranes, regulating mitochondrial calcium uptake.^70^ These findings suggest that traditional ER stress sensors may have a broader role beyond their established functions. Moreover, evidence suggests that ischemic stress, via multiple mechanisms, can stimulate the activity of the nuclear factor erythroid 2‐related factor (Nrf2),^71^, ^72^, ^73^ which participates in the elevation of endogenous antioxidant levels,^74^ the mitigation of apoptosis,^75^ and the enhancement of mitochondrial biogenesis.^76^ Furthermore, both the UPR and Nrf2 have been identified as pivotal contributors to the pathogenesis of cardiac hypertrophy and heart failure,^77^, ^78^ which represent significant and escalating public health concerns, particularly in industrialized societies with aging populations. Moreover, disturbances in interorganelle communication involving the ER and other cellular components in endothelial cells and cardiomyocytes may have a detrimental impact on CVDs.^79^ Given the crucial part that ER stress plays in the development of CVDs, there is an emerging opportunity to develop therapeutic strategies focusing on ER stress, particularly the maladaptive UPR, as a promising target for disease intervention. A comprehensive overview of a series of natural compounds that target the cardioprotective ER stress response has been provided in detail.^11^ Table 1 lists potential target proteins associated with ER stress and the UPR. It also shows how to manipulate ER stress and UPR associated modulators could help prevent or treat CVDs.

**TABLE 1:** ER stress in cardiovascular diseases.

Disease	Model	Manipulation	Phenotype	References
Cardiac hypertrophy	TAC	ATF6 cKO mice	Blunted cardiac myocyte hypertrophy and impaired cardiac function	80
	TAC	CHOP cKO mice	Less fibrosis, cardiac hypertrophy, and cardiac dysfunction	81
	Tunicamycin or TAC	Pak2‐cKO	Defective ER response, cardiac dysfunction, and cell death	82
	TAC	Nrf2 KO	Pathological cardiac hypertrophy, significant cardiac fibrosis and apoptosis, overt heart failure, and increased mortality	83
	TAC	AAV‐mediated Hrd1 knockdown and overexpression	The knockdown of Hrd1 exacerbated cardiac dysfunction and increased apoptosis and cardiac hypertrophy. Conversely, the overexpression of Hrd1 preserved cardiac function, decreased apoptosis, and attenuated cardiac hypertrophy	84
Heart failure	TAC	PERK KO mice	Protection against heart failure and lung remodeling induced by pressure overload	85
	TAC	IRE1α transgenic mice	Protect the heart against heart failure induced by pressure overload	86
	Nitrosative stress	Cardiomyocyte‐restricted overexpression of Xbp1s	Attenuated HFpEF phenotype	87
	TAC	Xbp1s cKO	Exacerbated the progression of heart failure	88
Ischemic heart disease	I/R	ATF6 KO mice	Increased myocardial I/R damage and decreased function	48
	I/R	ATF6–MER TG Mice	Induced ER stress gene and protected from I/R injury	89
	I/R	Conditionally activated ATF6 in the heart	Decreased ischemic damage and induced numerous genes, including MANF	90
	I/R	Xbp1 cKO mice	Developed more severe hypertrophy and manifested more robust fetal gene reactivation after I/R	91
	I/R	rAAV9–PERK	Decreased cellular apoptosis index in the cardiac tissue	92
Atherosclerosis	Human coronary specimens	Basal condition	Induced ER chaperones and death signals in atherectomy specimens obtained from the culprit lesions of patients with UAP	93
	Western‐type diet	ob/ob;Ldlr−/− mice ob/ob.Ldlr−/− mice	Increased activation of the UPR in response to free cholesterol loading in insulin‐resistant macrophages.	94
	TAC	DKO mice of systemically or endothelium‐specifically excising Nogo‐A/B gene on an ApoE−/− background	Resistant to the buildup of coronary atherosclerotic lesions and plaque rupture	95
	High cholesterol/high‐fat atherosclerotic mouse diet	ApoE−/− with a genetic deletion of IRE1α in the myeloid lineage	Enhanced foam cell formation	96
Hypertension	Chronic hypoxia	Notch3 mutant mouse	Severe pulmonary hypertension phenotype	97
	Ang II	Mice (basal condition)	Robust increased ER stress biomarkers and significant ER morphological abnormalities in the circumventricular subfornical organ	98
	Sugen/Hypoxia (SuHx) or monocrotaline (MCT)	Ntsr1 knockdown by AAV1 rat	Improved progression of PH in SuHx or MCT‐induced rat model	99
Vascular cell dysfunction	Ang II	TBCE cKO	Exacerbated endothelial dysfunction, aortic wall hypertrophy, and ER stress‐mediated VSMC hyperproliferation	100
	Oxygen‐induced retinopathy	Mice (basal condition)	Neuronal ER Stress is induced in hypoxic retinas and associated with revascularization failure	101
Autoimmune cardiomyopathy	β1‐ECII immunization	Rabbits (basal condition)	ER stress occurs in autoimmune cardiomyopathy induced by β1‐ECII peptide, enhanced by elevated NE	102
	ß1‐ECII immunization	Rabbits (basal condition)	Progressive left ventricular systolic dysfunction, dilation, and myocyte apoptosis	103
Metabolic cardiomyopathy	Fed HFD/HF for 16 weeks and treated with TUDCA	Rats	Improved metabolic parameters, and mitigated cardiovascular complications through increasing survival markers and decreasing ER and oxidative stress markers	104
	IL‐1 receptor antagonist, IL‐1Ra	STZ‐induced DCM rat	Activation of ER stress and lead to elevated myocytes apoptosis	105
Cardiotoxicity of anticancer drugs	Imatinib	Mice (basal condition)	Abnormalities in the mitochondria and accumulation of membrane whorls in the vacuoles and ER	106
	Oral dapagliflozin followed by DOX via intraperitoneal injection	STZ‐induced diabetic rats	Reduced cardiac fibrosis and significantly improved cardiac function	107

Abbreviations: AAV, adeno‐associated viral; Ang II, angiotensin; apoE, apolipoprotein E; β1‐ECII, the second extracellular loop of β1‐adrenoceptors; cKO, conditional knockout; DCM, diabetic cardiomyopathy; DKO, double knockout; DOX, doxorubicin; HFD/HF, high‐fat diet/high‐fructose; HFpEF, heart failure with preserved ejection fraction; I/R, ischemia/reperfusion; KO, knockout; Ldlr, lipoprotein receptor; MANF, mesencephalic astrocyte‐derived neurotrophic factor; NE, norepinephrine; ob/ob, obese; PH, pulmonary hypertension; rAAV9, recombinant adeno‐associated virus type 9; STZ, streptozotocin; TAC, transverse aortic constriction; TUDCA, tauroursodeoxycholate; UAP, unstable angina pectoris; VSMC, vascular smooth muscle cell.

### 4.2 Neurodegenerative diseases

Neurodegenerative diseases, such as Parkinson's disease (PD), Alzheimer's disease (AD), Huntington's disease (HD), and amyotrophic lateral sclerosis (ALS), are characterized by the gradual deterioration of neuronal integrity and functionality, constituting a leading cause of death globally. Abnormal protein aggregation is common in neurodegenerative diseases.^108^ Persistent activation of the UPR has emerged as a shared characteristic across diverse neurodegenerative diseases. A comprehensive review of the literature was conducted to examine the roles of ER stress and the UPR in the context of neurodegenerative disorders.^12^, ^109^, ^110^ Recent studies have begun to elucidate the specific mechanism for the disruption of ER homeostasis in neurodegenerative diseases, thereby shedding light on discrete points within the secretory pathway that undergo disruption. AD are characterized by the aggregation of the extracellular senile plaques of amyloid‐beta (Aβ) peptides and hyperphosphorylated tau (a microtubule assembly protein) within the brain.^111^, ^112^ The formation of Aβ is dependent upon the cleavage of the amyloid precursor protein (APP). This process can occur via two distinct pathways: the nonamyloidogenic pathway, which represents the normal processing route; and the amyloidogenic pathway, which represents an abnormal processing route.^113^ The cleavage of APP is initiated by signal peptidases during the translocation of APP from the ER to the Golgi apparatus, where it undergoes maturation. In the nonamyloidogenic pathway, α‐secretase cleaves APP, producing α‐fragments of APPs that lack toxicity.^114^, ^115^ However, alternative cleavage of APP by β‐secretase‐1 (BACE1) and γ‐secretase may result in the generation of neurotoxic Aβ in the amyloidogenic pathway.^116^ Moreover, amyloid pathology can result in impaired proteostasis.^117^ It is noteworthy that, in contrast to the amyloid plaques, the oligomers or intraneuronal Aβ may represent the most closely associated neurotoxic species.^118^, ^119^ Aβ(1–42) has been demonstrated to markedly elevate acetylcholinesterase (AChE) activity, thereby inducing ER stress and apoptosis.^120^ Concomitantly, elevated Aβ(1–42) production renders neuroblastoma cells more susceptible for ER stress‐induced toxicity.^121^ An ER‐specific apoptotic pathway has been identified as a key mechanism underlying the neurotoxic effects of prion proteins and Aβ peptide.^122^ Some calcium‐binding chaperones including GRP78 (referred to as BiP), endoplasmin (known as GRP94), calreticulin, and PDI have been observed to perform in an aberrant manner, thereby inducing ER stress.^123^ The protein levels of BiP/GRP78 are elevated in the temporal cortex and hippocampus of patients diagnosed with AD.^124^ It has been reported that GRP78 interacts with APP transiently and directly in the ER to regulate intracellular APP maturation and processing, facilitating its correct folding.^125^ Nevertheless, both oligomeric and fibrillar Aβ(1–42) do not induce BiP expression to a degree that can be discerned in a population of cells,^126^ indicating that elevated BiP expression in AD may be associated with specific cell types and temporal factors. Furthermore, the accumulation of tau protein results in aberrant interactions between proteins within the ER and key components of the ERAD pathway, which consequently impedes the progression of this pathway.^127^

It has been observed that ER stress has been activated in AD, which highlights the necessity to assess the contribution of additional mediators of the UPR in the pathophysiology of AD. The PERK and IRE1α signaling pathways are activated in the pyramidal cells of the hippocampus in patients with AD.^128^ The ATF4 protein and transcripts are observed with greater frequency in axons in the brain of patients with AD.^129^ These results come from studies using animal models of AD.^127^ Notably, PERK haploinsufficiency is sufficient to improve cholinergic neurodegeneration and memory deficits in AD mice,^130^ which also prevents BACE1 elevations and reduces amyloid‐β peptide levels and plaque burden in AD mouse model.^130^ This indicates that PERK may confer neuroprotective properties. IRE1 is a crucial mediator in the pathogenesis of AD. IRE1 activation has been demonstrated to correlate positively with the progression of AD histopathology in human brain tissue.^131^ The extent of IRE1α phosphorylation demonstrates a direct correlation with the Braak stage of pathology in individuals diagnosed with AD.^131^ An increased spliced XBP1 mRNA has been observed in the temporal cortex of patients diagnosed with AD.^132^ Genetic removal of the RNase domain of IRE1 in the nervous system lead to a notable reduction in the content of amyloid β oligomers, amyloid deposition, and astrocyte activation. The absence of IRE1 rescues learning and memory capacity, accompanied by improved long‐term potentiation (LTP) and enhanced synaptic function in AD mice.^131^ However, mice lacking XBP1 in the nervous system exhibites a specific impairment in the formation of contextual memory and LTP, whereas neuronal XBP1 overexpression leads  to an improvement in performance in memory tasks.^133^ The results of this study indicate that IRE1 plays an unexpected role in the development of AD, highlighting the possibility of other crucial compensatory pathways associated with IRE1. The role of ATF6 in neurodegeneration has been the subject of relatively little research. The basal levels of BiP within dopaminergic neurons are reduced in ATF6α KO mice.^119^ Mice lacking ATF6 exhibit heightened sensitivity to PD‐induced neurotoxins in animal models of PD.^134^, ^135^ In models of HD, the activity of ATF6 is suppressed, which contributes to the pathogenesis of the disease.^136^ These findings suggest that the function of the ATF6 pathway is crucial for maintaining proteostasis within this particular neuronal subset. Further research is required to determine the potential therapeutic efficacy of targeting this branch of the UPR signaling pathway for the treatment of diseases.

It has been observed that activation of the UPR in several non‐tau‐related diseases, including ALS and PD. In ALS, UPR activation has been documented in the spinal cord in sporadic cases,^137^ while in PD, UPR activation has been observed in the substantia nigra.^138^ Furthermore, in neurodegenerative disorders such as PD and AD, specific misfolded proteins resulting from genetic mutations have been identified. It has been demonstrated that mutations in presenilin‐1 may disrupt the equilibrium of the ER and subsequent activation of the UPR.^139^ However, findings that are contrary to this suggest that the loss of presenilin‐1 or the expression of its variants is insufficient to trigger UPR.^140^ Notably, a single molecule may serve as disparate functions in distinct pathological conditions. For example, the ablation of Xbp1 within the brain has been demonstrated to ameliorate symptoms associated with HD by triggering autophagy mechanisms that target the degradation of aggregated proteins,^141^ its function is opposite in AD.^133^ This indicates that the function of XBP1 in disease pathogenesis may be context‐dependent, thereby highlighting its adaptable nature. As research progresses, some novel early markers of ER stress associated with neurodegenerative diseases have been elucidated, including Sorcin.^142^ These findings indicate that our understanding of ER stress and UPR response in the development of AD is still on the way, with some complexities and controversies yet to be resolved. These contradictory findings highlight the necessity for further investigation to elucidate the intricacies of AD pathology and provide clearer directions for future therapeutic strategies.

### 4.3 Metabolic diseases

Metabolic diseases constitute a broad category of disorders characterized by abnormalities in the body's metabolism. The term “metabolism” is employed to delineate the intricate biochemical processes that are intrinsic to the sustenance of life in living organisms. These processes entail the conversion of food into energy and the synthesis of essential molecules that are requisite for the growth, repair, and maintenance of tissues. Metabolic diseases have emerged as significant threats to human health, precipitating the onset of severe chronic conditions including type 2 diabetes, obesity, and nonalcoholic fatty liver disease (NAFLD). It is noteworthy that although these diseases manifest distinct physiological and clinical manifestations, they exhibit shared pathological characteristics, notably intracellular inflammation and stress induced by metabolic disruptions originating from excessive nutrient intake, which is often exacerbated by contemporary sedentary lifestyles. Elevated levels of fatty acids (FAs) and glucose in cellular and organ systems foster conditions of lipotoxicity and glucotoxicity. The molecular mechanisms of lipotoxicity and glucotoxicity in NAFLD have been summarized in detail in the review previously.^143^

Metabolic stress can be defined as the phenomenon whereby cells receive an amount of fuel that is either in excess or deficient in their energy requirements. The primary nutrients that can harm cells by affecting the ER are free FAs and glucose. The oxidation of free FAs released from triglycerides derived from dietary fats is the primary means by which peripheral tissues meet their energy requirements. In the event of an excess of FAs, these are stored in adipocytes for utilization during periods of fasting. However, circulating FAs will rise if the supply of FAs exceeds the capacity of adipose tissue for storage. This excess can result in the accumulation of lipids in cells and tissues that lack the necessary mechanisms for processing and storing them, leading to cellular damage. Recent research indicated that the deleterious effects of saturated FAs, such as palmitate (C16:0), may be associated with their excessive incorporation into membrane structures including the ER. This reduction in the presence of essential lipids, such as sphingomyelin and cholesterol, is necessary for optimal ER function. Consequently, this leads to lipotoxicity in pancreatic β‐Cells.^144^ Additionally, elevated levels of free cholesterol in the circulation can induce ER lipotoxicity by modifying the composition and stability of the ER membrane.^145^ The ER is sensitive to cholesterol levels and plays a vital role in regulating cholesterol metabolism. This is achieved through ER‐resident transcription factors, such as nuclear factor erythroid 2 related factor‐1 (Nrf1; also known as Nfe2L1), which maintain cholesterol homeostasis.^146^ Conversely, the chronic consumption of diets with a high sugar content can result in glucotoxicity, which causes structural and functional damage to cells, particularly affecting organs such as the blood vessels, kidneys, retina, and peripheral nerves. In addition to glucose, elevated levels of fructose or sucrose have also been demonstrated to induce insulin resistance in rodent models.^147^ It has been demonstrated that glucotoxicity enhances N‐linked protein glycosylation, although its effects on the ER are less evident in comparison with those of lipotoxicity.^143^ In contrast, glucotoxicity predominantly induces oxidative stress, which can subsequently precipitate ER stress.^148^ Furthermore, lipotoxicity and glycotoxicity may act in synergy, a phenomenon known as glucolipotoxicity. In this context, glucose is observed to amplify the toxic effects of lipids.^149^ Nevertheless, the precise mechanism underlying this synergy  elusive. Moreover, the ER and mitochondria work together as sensors of nutrient excess and coordinators of the body's response to metabolic stress. Cells that are subject to glucotoxicity and/or lipotoxicity can activate a range of intracellular stress signaling pathways, encompassing oxidative stress and ER stress responses, to mitigate metabolic stress.^150^

The disruption of UPR pathways in genetically modified mice has demonstrated that the maintenance of ER homeostasis plays a regulatory role in glucose and lipid metabolism (Table 2). Accumulating evidence suggests that ER stress and the UPR signaling pathways play a role in the development and progression of metabolic diseases such as NAFLD^151^ and type 1 diabetes.^152^ The PERK branch of the UPR pathways plays an important role in regulating the rate of protein synthesis, particularly proinsulin and its translocation into the ER for proper protein folding. Mutations in the PERK gene have been associated with Wolcott‐Rallison syndrome in humans.^153^ A *Perk* knockout mutation in mice leads to the loss of insulin‐secreting beta cells and the development of diabetes mellitus.^154^ From a mechanical perspective, the loss of *Perk* function does not result in uncontrolled protein synthesis, rather it impairs ER‐to‐Golgi anterograde trafficking.^155^ PERK regulates proinsulin proteostasis by modulating the activity of ER chaperones, including BiP and ERp.^156^ Furthermore, IRE1–XBP1 pathways have been identified as a key player in the pathogenesis of diabetes. The IRE1 pathway is found to be specifically activated in pancreatic beta cells of rats infected with viruses at early stage, preceding the development of insulitis. This resulted in the apoptosis of beta cells in a viruses‐induced rat model of type 1 diabetes.^157^ Mice with β cell‐specific Ire1α cKO exhibited the hallmark diabetic phenotype, including impaired glycemic control and defects in insulin biosynthesis. The deletion of Ire1α in pancreatic β cells and insulinoma cells resulted in a reduction in insulin secretion, a decline in the levels of both insulin and proinsulin within cells, and a decrease in the oxidative folding of proinsulin. This was accompanied by a reduction in the expression of five PDIs: PDIR, P5, PDI, ERp44, and ERp46.^158^ In addition, nonreceptor ABL tyrosine kinases have been demonstrated to enhance the enzymatic activities of IRE1α, thereby potentiating ER stress‐induced apoptosis. The inhibition of the ABL–IRE1α pathway with selective IRE1a kinase inhibitors or imatinib (tyrosine kinase inhibitor）has been shown to reverse autoimmune diabetes in mice,^159^ indicating the ABL–IRE1α axis as a potential drug target for treating autoimmune diseases. Nevertheless, the deletion of IRE1α before insulitis resulted in transient dedifferentiation of β cells, leading to a notable decrease in islet immune cell infiltration and β cell apoptosis.^160^ This finding provides a promising basis for developing a novel preventive strategy for type 1 diabetes mellitus in individuals at high risk. More importantly, subthreshold ER stress is found to drive insulin demand‐induced β cell proliferation through the activation of ATF6.^161^ These findings indicate the possibility of a correlation between ER dysfunction and insulin signaling in the context of diabetes. Prolonged metabolic stress can trigger both ER stress and oxidative stress, which is often accompanied by inflammation.^162^ These conditions are characterized by widespread dysfunction across multiple organs, including the liver, muscles, adipose tissue, brain, and pancreas. However, some mechanisms related to ER stress are shared among these organs, others are specific to certain cell types.

**TABLE 2:** ER stress in metabolic diseases.

Disease	Model	Manipulation	Phenotype	References
Diabetes	STZ/nicotinamide	Male Wistar rats	The induction of ER stress and a notable reduction in the expression of glucose transporter proteins	163
		*Perk* mutant mice	Hyperglycemia and reduced serum insulin levels	164
		*Perk* cKO	The Islets of Langerhans undergo progressive degeneration, resulting in a loss of insulin‐secreting beta cells and the development of diabetes mellitus. Subsequently, a loss of glucagon‐secreting alpha cells occurs	154
		*Ire1α* cKO	Glycemic control is impaired, and there are defects in the biosynthesis of insulin	158
Insulin resistance				164
	HFD	*Pkr* KO	Glucose metabolism and insulin sensitivity	165
	Treadmill running	ATF6α KO mice	The process of recuperation from muscle damage sustained during exercise is not optimally efficient	166
	One bout of equal‐distance pair running	PGC‐1α muscle‐specific transgenic mice	Defective in its ability to upregulate ER chaperones and is susceptible to exacerbated ER stress following repetitive exercise challenges	167
Obesity				164
	HFD	Myeloid‐specific IRE1α abrogation	Reversed the imbalance in white adipose tissue caused by a high‐fat diet and prevented obesity, insulin resistance, high cholesterol, and liver damage	167
	HFD	CerS6 cKO Conditional deletion of CerS6 in hypothalamic neurons	Prevents weight gain and improves glucose metabolism	168
	Cold exposure	Ern1AKO Ern1BKO	Adipocyte IRE1α ablation results in the promotion of cold‐induced WAT browning	169
Hyperlipidemia	16 h fast	The hepatocyte‐specific *Ire1α* null mice	Accentuated hepatosteatosis without affecting De Novo lipogenesis	151
	Injection of poly(I:C)	Mice lacking XBP1 in the liver (Xbp1Δ)	Dramatic decreases in plasma triglyceride, cholesterol, and free FA	170
		*Ire1α* cKO	Modest hypolipidemia	171
Nonalcoholic fatty liver disease (NAFLD)	High‐fat WD	Hepatocyte‐specific nuclear activation (NFATc1^c.a^) or depletion (NFATc1^Δ/Δ^)	NFATc1 activation drives FA‐induced NASH and fibrosis	172
	Fructose on HFD	Liver‐specific knockdown of KHK in mice	Improved metabolic dysfunction	173
	Chow‐diet fed	FOXA3 KO mice	Alleviation of lipid accumulation in hepatocytes and a concomitant decrease in liver triglyceride levels	174
	HFD	Liver‐specific deletion of ENDOG	Repressed HFD‐induced liver lipid accumulation	175
	HFD	Casp2‐deficient MUP‐uPA mice	Prevent diet‐induced steatosis and the progression of NASH in mice with a predisposition to ER stress.	176

Abbreviations: BKO, brown adipose tissue (BAT)‐specific knockout; AKO, adipose‐specific knockout; NASH, nonalcoholic steatohepatitis; TG, triglyceride; WAT, white adipose tissue; WD, western diet.

### 4.4 Autoimmune diseases

Autoimmune diseases are a group of disorders that are defined by aberrant immune responses directed against the cells and tissues of the body. In such circumstances, the immune system is unable to distinguish “self” and “non‐self,” resulting in an attack on healthy tissues. This results in the development of inflammation, tissue damage, in many cases, a loss of functionality in the affected organs. ER stress plays a vital role in the pathogenesis of autoimmune diseases, including Crohn's disease, inflammatory bowel disease (IBD), and rheumatoid arthritis. The available experimental evidence indicates that abnormalities in the UPR may contribute to the development of autoimmune diseases. In murine models of HLA‐B27‐associated ankylosing spondylitis^177^ and inclusion body myositis,^178^ misfolded proteins have been demonstrated to act as autoantigens. When the UPR is excessively activated and misfolded proteins accumulate, it has the potential to trigger autoimmunity. Proteins associated with the UPR may potentially serve as autoantigens, as indicated by the detection of autoantibodies specific to BiP in individuals with rheumatoid arthritis^179^ and a Sjögren's syndrome mouse model.^180^ In nonimmune cells, dysfunctional UPR pathways have the potential to contribute to the development of autoimmunity by overwhelming the normal mechanism of immune tolerance. This scenario may occur in tissues such as the intestinal epithelium, the vascular endothelium, and the central nervous system. The results of studies conducted on mice have demonstrated that the malfunctioning of UPR pathways in specific tissues can result in the onset of various pathological conditions, including experimental autoimmune encephalomyelitis (EAE), colitis, and atherosclerosis. To illustrate, the intestinal epithelium serves as a physical barrier against various intestinal microorganisms, the majority of which constitute the normal bacterial flora. It is hypothesized that issues with the permeability of intestinal epithelial cells may be a contributing factor in the development of inflammatory colitis, as observed in human IBD. At present, the intestine, particularly in the intestinal epithelial cells, is the only known site of IRE1β expression.^181^ It is noteworthy that the expression of BiP is increased in intestinal epithelial cells in mice with *Ire1β*, suggesting elevated basal levels of ER stress in these cells.^181^ Furthermore, in a colitis mouse model, the onset of intestinal inflammation occurs 3−5 days earlier in *Ire1β* deficiency mice compared with control mice.^181^ It seems reasonable to suggest that defective UPR in intestinal epithelial cells in these mice may result in increased epithelial permeability, thereby exacerbating colitis. Moreover, an augmentation in the ERAD pathway may facilitate the evasion of UPR‐induced apoptosis or confer a survival advantage upon autoreactive cells, a phenomenon known as ‘hyper‐ERAD’. This concept is supported by evidence from at least one mouse model of autoimmunity. Synoviolin (SYVN1), an E3 ubiquitin ligase resident in the ER, is involved in the ERAD pathway. The expression of SYVN1 was markedly elevated in patients with newly diagnosed conditions in comparison with healthy controls.^182^ The overexpression of SYVN1 throughout the body in mice results in the spontaneous development of peripheral arthropathy, which is characterized by bone destruction and synovial hyperplasia.^183^ The results indicate that SYVN1 overexpression results in a “hyper‐ERAD” state, which may be a contributing factor in the development of arthritis. This state may enhance the functional efficiency of inflamed SYVN1 or autoreactive cells by alleviating ER stress or reducing susceptibility to apoptosis induced by ER stress. It is noteworthy that recent research has demonstrated that the expression of SYVN1 promoted the degradation of IRE1, potentially protecting cells from IRE1‐mediated apoptosis in mouse synovial cells.^184^ Nevertheless, it remains unclear whether the UPR was hyperactive in the IRE1‐deficient cells. The persistence of inflammation can result in tissue injury, which in turn facilitates the progression of affected tissues.

The relationship between inflammatory signaling and the UPR in autoimmune diseases remains incompletely understood, despite some existing evidence. A case in point is IBD. The impairment of ER function by the loss of AGR2, a member of the PDI family, has been observed to result in the development of terminal ileitis and colitis in mice.^185^ Additionally, AGR2 has been recognized as a potential genetic risk factor for the development of IBD in humans.^186^ It appears that exaggerated IRE1α signaling may be a driving force in Crohn's disease‐like ileitis mouse model, whereas ATG16L1‐dependent autophagy seems to exert a restraining influence on IRE1α signalling.^187^ Exaggerated IRE1α signaling appears to drive Crohn's disease‐like ileitis in mice, whereas ATG16L1‐dependent autophagy restrains IRE1α signaling pathway.^188^ As a result, IRE1α is found to accumulate in the intestinal crypts of animals that express the IBD risk allele of the autophagy protein ATG16L1 (T300A).^188^ In contrast, the loss of an intestinal cell‐specific paralog, IRE1β, has been shown to increase the likelihood of developing dextran sulfate sodium (DSS)‐induced colitis in mice.^189^ The chemical chaperones including 4‐PBA and TUDCA have been demonstrated to improve DSS‐induced colitis in mice with defects in UPR signaling.^190^ The utilization of more selective pharmacological agents in future studies may prove to be informative, as demonstrated in other models of inflammatory signaling. To illustrate, NOD1 and NOD2 are pattern recognition receptors that assist the body in responding to stress.^191^ IL‐6 is inhibited by KIRA6 (an IREα kinase inhibitor) in response to thapsigargin, indicating that IRE1 kinase activity may play a specific role in this pathway.^191^

### 4.5 Fibrotic diseases

Tissue fibrosis represents a pathological feature in a multitude of chronic diseases. It comprises progressive architectural alterations, characterized by the accumulation of extracellular matrix (ECM) and collagen, which frequently occur alongside organ dysfunction and eventual failure. Tissue fibrosis typically follows an acute or gradual injury, with various potential triggers including tissue infection/inflammation, ischemia, and toxic exposures. The existence of a multitude of potential antecedent events suggests that there are shared molecular mechanisms that contribute to the development of tissue fibrosis. The issue of protein folding and quality control is present in many chronic fibrotic conditions. The results of studies examining the etiology of inherited forms of fibrotic diseases have indicated that the protein quality control system may exert a direct influence on the process of tissue fibrosis. The aforementioned diseases include familial chronic kidney disease, the familial form of idiopathic pulmonary fibrosis (IPF), and α1‐antitrypsin‐related cirrhosis. In each instance, genetic mutations are identified that resulted in the incorrect production of proteins. This results in stress within the ER, which then activates the UPR signaling cascade. Further research has demonstrated that the UPR signaling pathway is involved in the pathogenesis of progressive fibrotic disorders affecting various organs (Table 3).

**TABLE 3:** ER stress in fibrotic diseases.

Disease	Model	Manipulation	Phenotype	References
Pulmonary disease	Basal condition	Alveolar type II cell‐specific Grp78 knockout mice	Developed fibrosis	192
	Basal condition	PINK1 KO mice	Exhibited comparable mitochondrial dysmorphia and dysfunction in AECIIs, rendering them susceptible to apoptosis and the onset of lung fibrosis	193
	Crystalline silica (CS)	Pharmacological inhibition of IRE1α	Improved pulmonary function and a delay in the development of CS‐induced lung fibrosis	194
	Mechanical ventilation	WT	Promoted ASK1‐mediated ER stress and extracellular vesicle (EV) release from alveolar epithelial cells	195
Hepatic fibrosis	CCl_4_‐induced liver fibrosis	Txndc5^cKO^ (hepatic stellate cells) Txndc5^Hep‐cKO^ (Hepatocytes)	Improved in liver fibrosis	196
	Tunicamycin	*Fxr* KO	Worsens ER stress‐induced inflammasome activation and hepatocyte injury	197
	Bile duct ligation	Sestrin2 KO	Exacerbated cholestatic liver injury	198
	Basal condition	Mic19 liver‐specific knockout mice	NASH and liver fibrosis	199
Chronic kidney disease	Unilateral ischemia–reperfusion injury (UIRI)	FITC‐labeled Klotho‐derived peptide (KP1) tail vein injection	Preserved renal functions and ameliorated renal fibrosis in UIRI mice	200
	Unilateral ureteral obstruction	DOX ‐inducible widespread Rtn1a knockdown	Alleviated ER stress and renal fibrosis in mice with unilateral ureteral obstruction; mitigated ER stress, glomerular hypertrophy, proteinuria, and mesangial expansion in diabetic mice	201
	STZ	COX‐2 KO in hematopoietic cells or macrophages	Developed severe diabetic nephropathy	202
	Ang II	Fatostatin intraperitoneally	Attenuated Ang II‐induced renal fibrosis	203

Abbreviations: AECII, type II alveolar epithelial cell; CS, cigarette smoking; COX‐2, cyclooxygenase‐2; FITC, fluorescein isothiocyanate; UIRI, unilateral ischemia reperfusion injury; WT, wild type.

The death of lung and other organ epithelial cells can result in fibrosis due to the inhibition of re‐epithelialization following injury or impairment of the epithelial barrier functions. ER–mitochondrial interactions are of pivotal importance in the regulation of cellular bioenergetics and cell death signaling, facilitated by the sequestration of intracellular calcium.^204^, ^205^, ^206^ Additionally, ER stress can instigate proapoptotic signaling via each of the three branches of the UPR cascades, as previously discussed in the section on cell fate determination under UPR. Moreover, sustained inflammation can lead to tissue damage and aberrant repair mechanisms, which facilitate fibrotic remodeling of affected tissues. The aggregation of proteins within the ER can facilitate the binding of NF‐κB to DNA, thereby regulating gene expression that is dependent on the κB factor.^207^ IKK and IRE1α form a complex through the adapter protein TRAF2 in response to ER stress. Further studies have shown that ER stress reduces the expression of TRAF2, which stops TNF‐α from activating NF‐κB and JNK. This makes TNF‐α more powerful at causing apoptosis.^208^ Furthermore, evidence indicates that PERK activation leads to increased NF‐κB activity, which can be attributed to the suppression of IκBα translation.^209^ Nevertheless, the activation of inflammatory signaling appears to be contingent on the context and cell type. This is evidenced by the fact that NF‐κB activation is not universally observed in all ER stress models. Indeed, in some systems, there is even suppression of LPS and TNF‐α‐induced NF‐κB activation,^210^ along with the inhibition of NF‐κB‐driven cytokine production.^211^ Moreover, evidence indicates that ER stress affects the number and phenotype of immune and inflammatory cells, with a particular focus on macrophages. The expression of CHOP has been demonstrated to be altered in patients diagnosed with IPF. CHOP deficiency protects against bleomycin‐induced IPF in mice by reducing the production of M2 macrophages.^212^ Furthermore, research has indicated that ER stress can enhance the suppressive phenotypes of immune cells. The activity of a PERK‐signaling cascade in macrophages is increased by helper T cell 2 cytokines interleukin‐4 and the tumor microenvironment (TME), thereby promoting immunosuppressive M2 activation and proliferation.^213^ Furthermore, defects in efferocytosis have been recognized as a potential contributing factor to the development of various inflammatory diseases. A recent research study employes a strategy to enhance efferocytosis, designated as the “chimeric receptor for efferocytosis” (CHEF), to generate BELMO and TELMO. The results demonstrate that phagocytes expressing CHEF exhibites a significant enhancement in efferocytosis. In murine models of inflammation, the expression of BELMO has been demonstrated to attenuate the severity of colitis, hepatotoxicity, and nephrotoxicity. Mechanistic studies have demonstrated that BELMO enhances the presence of enzymes and chaperones within the ER, thereby mitigating the toxicity associated with protein folding. This mechanism is subsequently confirmed in a model of renal ischemia‐reperfusion injury induced by ER stress. Moreover, the administration of TELMO following the onset of kidney injury is observed to significantly reduce fibrosis.^214^ These findings collectively indicate that enhanced efferocytosis may function as a potential strategy for mitigating inflammation in fibrotic disease.

It has been established that epithelial cell dysfunction plays a pivotal role in the etiology of diffuse parenchymal diseases, including IPF. The role of alveolar epithelial cell quality control in the pathogenesis of pulmonary fibrosis has been reviewed in reference.^215^ The deletion of GRP78 has been observed to manifest characteristics of IPF, including weight loss, lung inflammation, spatially heterogeneous fibrosis, and mortality. This latter feature is characterized by the presence of hyperplastic alveolar type 2 cells, fibroblastic foci, and increased susceptibility in old and male mice.^192^ Therefore, it may be proposed that modulation of ER stress and chaperone function represents a promising avenue for developing therapeutic approach to pulmonary fibrosis. Moreover, evidence indicates that ER stress may induce epithelial‐to‐mesenchymal transition (EMT) in alveolar epithelial cells,^216^ potentially through Src‐dependent pathways.^209^ The EMT has been demonstrated to be promoted by the IRE1–XBP1 signaling pathway via the mediation of Snail expression in pulmonary fibrosis.^217^ ER stress impedes the function of Wnt‐driven epithelial stem/progenitor cells, specifically in the downstream phase following the nuclear localization of β‐catenin.^218^ This indicates that ER stress may impede the capacity of local progenitor cell niches to undergo renewal.

### 4.6 Viral infection

Viruses are highly adept at molecular manipulation and have evolved to flourish and survive in all species. The continued success of viruses is contingent upon their capacity to subvert host cell defense mechanisms, thereby ensuring their survival, replication, and proliferation. Following infection, the virus is required to synthesize proteins rapidly. Furthermore, nonenveloped viruses require proteins and lipids for replication. It is therefore unsurprising that viruses cause ER stress and manipulate the UPR signaling pathway. To illustrate, the SARS‐CoV‐2 ORF3a protein has been observed to induce RETREG1/FAM134B‐dependent reticulophagy and to elicit a series of inflammatory responses and ER stress during SARS‐CoV‐2 infection.^28^ SARS‐CoV‐2 nonstructural protein 6 has been demonstrated to result in autophagy induced by ER stress, which lead to the degradation of STING1.^219^ Pharmacological reprogramming of ER stress pathways represents a potential strategy for the suppression of coronavirus replication (CoVs). Thapsigargin can efficiently inhibit the replication of coronaviruses (MERS‐CoV, HCoV‐229E, SARS‐CoV‐2) in primary differentiated human bronchial epithelial cells.^220^ Nevertheless, the relationship of UPR signaling pathway between the host and the virus is complex and not yet fully elucidated.

It seems plausible to suggest that the three branches of the UPR could be used as a kind of antiviral defense. The PERK pathway of the UPR may serve as a host antiviral defense, as this kinase is similar to and functions in a manner analogous to the double‐stranded RNA‐activated protein kinase PKR, which is recognized as one of the defenses against viruses. For example, mouse embryonic fibroblasts (MEFs) derived from PERK knockout mice have been observed to exhibit heightened susceptibility to vesicular stomatitis virus (VSV)‐mediated apoptosis relative to their PERK‐intact counterparts. It seems that the VSV can replicate more effectively in PERK knockout MEFs because it cannot slow down the production of viral proteins, which is caused by impaired eIF2α phosphorylation.^221^ In another mouse model of tumor growth, deficiencies in translational regulation via the alpha subunit of eIF2 have been demonstrated to impede antiviral activity and facilitate the malignant transformation of human fibroblasts..^222^ It's worth mentioning that the porcine hemagglutinating encephalomyelitis virus (PHEV) has been seen to cause ER stress and activate the UPR in vivo and in vitro. Moreover, the PERK/PKR‐eIF2α pathway has been shown to stop PHEV from replicating by reducing global protein translation and encouraging stress granule (SG) formation.^223^ These findings collectively confirmthat PERK exerts its antiviral effects through translation blocking.

IRE1α is the most well‐preserved branch of the UPR, exhibiting both RNase and kinase activities. It has been demonstrated that IRE1α is activated in infectious bronchitis virus (IBV)‐infected cells and plays a role in their survival during coronavirus infection.^224^ The expression of IRE1α is observed to increase during the course of herpes simplex virus type 1 (HSV‐1) infection. The elevation in RNase activity or the suppression of the kinase activity of IRE1α results in a reduction in viral replication in endometrial cancer cells HEC‐1‐A. This indicates that the kinase activity of IRE1α is conducive to viral replication, whereas the RNase activity is deleterious. Further evidence indicates that the JNK pathway is activated by the kinase activity of IRE1α, which enhances viral replication. In conclusion, the evidence presented here indicates that IRE1α plays an important role in the replication of HSV‐1, with its RNase and kinase activities exerting differential effects during viral infection.^225^ Moreover, the ATF6 branch has been shown to play a crucial role in reducing virus titers during viral infection. In a study conducted by Chen et al,^226^ it demonstrates that autophagy is activated by the Japanese encephalitis virus (JEV) in neuronal cells via the XBP1 and ATF6 ER stress sensors.

Nevertheless, as with the heads and tails of a coin, it can be posited that ER stress and the UPR signaling pathways may not help to protect from viral infection. Alternatively, viruses may exploit these processes to facilitate their replication. It has been demonstrated that the human coronaviruses SARS‐CoV‐2 and HCoV‐OC43 require the host proteins IRE1α and XBP1 for robust infection.^227^ A variety of strategies and mechanisms are employed by viruses to exploit the UPR branches. In cells infected with SARS‐CoV‐2 and in bystander cells that are not infected, levels of NUAK2 are found to be elevated as a consequence of IRE1α‐dependent processes. This promotes the spread of the virus and facilitates virion binding to bystander cells, thereby enhancing the infection process.^228^ PERK plays a vital role in the translation of alphavirus nonstructural proteins and has been demonstrated to impact multiple RNA viruses, rendering it an appealing target for the development of antiviral agents.^229^ VSV and hepatitis C virus (HCV) have been observed to accelerate the degradation of IFNAR1 via the induction of PERK‐dependent IFNAR1 degron phosphorylation.^230^ Infection with the Japanese encephalitis virus (JEV) activates the ER stress sensor PERK in neuronal cells and mouse brains. Activation of PERK has been demonstrated to induce apoptosis via the PERK–ATF4–CHOP apoptosis pathway in the context of JEV infection. Among the JEV proteins (E, prM, NS1, NS2A, NS2B, and NS4B), only NS4B has been demonstrated to activate PERK. Furthermore, evidence suggests that PERK plays a role in the apoptosis and encephalitis induced by JEV and NS4B.^231^ The oncogene latent membrane protein 1 of Epstein‐Barr virus（EBV）has been demonstrated to activate PERK and the UPR, thereby driving its synthesis.^232^ A consequence of Seneca Valley Virus (SVV) infection is the degradation of STING via PERK and ATF6‐mediated reticulophagy, which may represent an immune escape strategy of SVV.^233^ Porcine epidemic diarrhea virus has been observed to manipulate the ER to perturb its redox homeostasis. This is achieved via the PERK–CHOP–ERO1α–ROS axis, which is exploited by the virus to facilitate its replication.^234^ Classical swine fever virus infection induces autophagy through the activation of the IRE1 and PERK pathways, which facilitate viral replication in cultured cells.^235^ The activation of IRE1α by the HCV protein NS4B in XBP1‐proficient cells has also been observed to confer resistance to apoptosis and to promote viral replication.^236^ The adenovirus E3‐19K glycoprotein has been observed to elicit a specific response in the UPR sensor IRE1α within the ER, while other UPR sensors remain unresponsive. The E3‐19K lumenal domain has been demonstrated to activate the IRE1α nuclease, which in turn initiates the splicing of XBP1 mRNA. XBP1s has been observed to bind to the viral E1A‐enhancer/promoter sequence, thereby enhancing E3‐19K levels, E1A transcription, and lytic infection.^237^ These findings indicates that the virus is specific in its remodeling of the ER to form replication organelles, which ultimately leads to ER stress and UPR.

During the confrontation between the virus and the host, a variety of molecules are involved in regulating the UPR, which ultimately determines the outcome of this battle. The loss of TRIM29 has been observed to reduce viral myocarditis in male mice by attenuating the PERK‐driven ER stress response, indicating that targeting the TRIM29–PERK axis may be a potential strategy for mitigating disease severity.^238^ PDIs exert a negative regulatory effect on ebolavirus glycoprotein expression, thereby balancing the viral life cycle by maximizing infection while minimizing the cellular effect. It is proposed that ebolaviruses hijack the host protein folding and ERAD machinery to enhance fitness through reticulophagy during infection.^239^ The inhibition of USP14 has been demonstrated to influence the proliferation of alphaherpesviruses by triggering the degradation of the viral VP16 protein via ER stress‐induced selective autophagy.^240^ The findings offer a novel perspective on potential therapeutic targets for the treatment of virus‐induced infectious diseases.

### 4.7 Cancer

Due to unfavorable environmental factors, metabolic changes, and oncogenic pathways, cancer cells are exposed to ER stress within the TME. It is frequently observed that transformed cells exploit the UPR signaling pathways as a significant adaptive mechanism that fuels their aggressive behavior. Furthermore, evidence suggests that the UPR plays a role in the development of chemoresistance in cancer cells. Consequently, comprehensive UPR signatures have been demonstrated to hold prognostic significance in several cancers, including breast, bladder, and osteosarcoma.^241^, ^242^, ^243^ This section presents the latest research on the role of ER stress response in cancer cells.

The IRE1α–XBP1s pathway has been demonstrated to promote some processes associated with proliferation of cancer cell, epithelial‐to‐mesenchymal transition (EMT), and drug resistance.^244^ There are present molecular cross‐talks between hypoxic ER stress, HIF1α pathway and LRP6/β‐catenin signaling.^245^ XBP1 has been demonstrated to promote the development of triple‐negative breast cancer through HIF1α pathway.^246^ Furthermore, hypoxia‐induced ER stress results in the downregulation of WNT/β‐catenin signaling, which in turn leads to XBP1s‐mediated transcription of prosurvival hypoxia response genes in colon cancer cells.^247^ Furthermore, evidence indicates that IRE1α–XBP1s contributes to the maintenance of stemness in ovarian cancer stem cells (CSCs). The transcription factor FOXK2 binds to a regulatory element of ERN1, resulting in elevated levels of IRE1α and facilitating XBP1s‐mediated induction of genes associated with stemness in human ovarian CSCs. The concurrent inhibition of ER stress and oxidative stress has been demonstrated to effectively repolarize M2 tumor‐associated macrophages (TAMs) under hypoxic conditions.^248^ Tumor growth is delayed by the introduction of XBP1s into germinal center B cell‐like diffuse large B‐cell lymphoma cells in a xenograft model, suggesting a paradoxical antitumor effect of IRE1α–XBP1s in this particular cancer type. This study highlights the potential for variability in the response of different cancer types and subtypes to IRE1α inhibitors. Therefore, it is recommended that further investigations be undertaken to determine the range of cancer types that could potentially benefit from the therapeutic modulation of the UPR. Recent research indicates that RIDD may also be involved in the progression of tumors. In luminal breast cancer, IRE1α has been observed to degrade several protumourigenic microRNAs (miRNAs), including miR‐374a, miR‐3607, and miR‐96. This results in the upregulation of the RAS oncogene GTPase, RAB3B245.^249^ Conversely, the IRE1α–XBP1s pathway has been observed to regulate both pro‐ and antitumor effects by modulating tumor invasion, macrophage infiltration, and angiogenesis in glioblastoma. A high XBP1s and low RIDD have been linked to poor patient survival, indicating that the equilibrium between these two pathways may possess prognostic value.^249^

PERK exerts a dual regulatory effect on both pro‐ and antitumor responses, contingent upon the duration and intensity of ER stress. The transient activation of PERK in cancer cells triggers the prosurvival PERK–ATF4–NRF2 pathway.^250^ The contradictory effect of PERK on cancer cell survival is attributed to the expression of miR‐211. PERK–ATF4 induces miR‐211 during ER stress, which suppresses the stress‐induced expression of proapoptotic CHOP. Nevertheless, miR‐211 expression is suppressed, which subsequently results in CHOP‐mediated apoptosis when PERK is markedly activated.^59^ A PERK‐specific inhibitor has been demonstrated to impede metastatic progression by constraining the ISR‐dependent survival of quiescent cancer cells.^251^ In addition, intrinsic PERK activation plays a role in the immunoinhibitory effects of tumor‐associated myeloid‐derived suppressor cells.^252^ The potential influence of type I interferon receptor expression on the prosurvival versus proapoptotic effects of PERK in tumor cells has been discussed and requires further investigation.^253^ This finding may assist in elucidating the dual role of eIF2α phosphorylation associated with PERK in promoting cell survival and metastasis, as well as inducing immunogenic cell death in tumor cells following treatment with specific anticancer agents.

The role of intrinsic ATF6 represents an avenue for further research in cancer cells, which remains largely uninvestigated. The majority of current researches on ATF6 in cancer have concentrated on how its prosurvival function can be harnessed to promote tumor growth, malignant progression, and chemoresistance, while also influencing oncogenic microbial dysbiosis and autophagy regulation. ATF6 is associated with a reduction in disease‐free survival in patients with colorectal cancer. Sustained intestinal activation of ATF6 in the colon has been demonstrated to facilitate dysbiosis and microbiota‐dependent tumor formation in mice with intestinal epithelial cell‐specific expression of the active form of ATF6.^254^ In vitro studies suggest that ATF6 may promote cell growth, migration, and autophagy in cervical cancer through ER stress and MAPK signaling pathways.^255^ However, further extensive research is required to improve our understanding of the function of ATF6 in cervical cancer in vivo and the clinical context. ER stress‐related ATF6 has been shown to upregulate CIP2A, which contributes to colon cancer prognosis.^256^ Similarly, mutant p53 has been observed to increase the resilience of cancer cells to ER stress through the sustained activation of ATF6.^257^ Gastrointestinal stromal tumors have been reported to evade ER quality control and ER stress‐mediated cell death by activating the UPR and using the ER quality control‐free Golgi apparatus to initiate signaling pathways.^258^ In addition, ATF6 signaling has been observed to induce the awakening of slow‐cycling of non‐small cell lung cancer cells by upregulating epidermal growth factor and stimulation of angiogenesis.^259^

Interactions between cellular stress and oncogenic signaling have been demonstrated. The T‐cell leukaemia/lymphoma 1 (TCL1) oncoprotein has been observed to interact with XBP1 in chronic lymphocytic leukemia (CLL), and the inhibition of XBP1s expression resultes in a reduction in the progression of CLL.^259^ In addition, the process of detachment of ductal carcinoma in situ (DCIS) cell detachment from the ECM has been shown to induce significant levels of cellular stress. Activation of PERK during ECM detachment has been observed to reduce levels of toxic ROS and promote autophagy, allowing DCIS cells to escape detachment‐associated cell death (anoikis).^260^ However, it is essential that stress‐relieving mechanisms, such as those mediated by PERK, are in place to ensure the survival of transformed cells. To illustrate, in chronic myeloid leukemia (CML), the BCR–ABL fusion protein induces ER stress, which in turn activates the PERK–eIF2α pathway. PERK activation has been shown to sensitize CML cells to imatinib treatment.^261^ Similarly, drugs that target the histone methyltransferase G9A induce oxidative stress, which is counteracted by the activation of PERK and NRF2. This allows acute myeloid leukemia cells to evade cell death induced by G9A inhibitors.^262^ Conversely, evidence suggests that ER stress may contribute to the development of hepatocellular carcinoma in a high‐fat‐diet‐induced inflammatory environment. This is based on the observation that ER stress generates preoncogenic cells that ultimately develop into hepatocellular carcinoma. This indicates that ER stress may function as an oncogenic role in hepatocellular carcinoma.^263^ Moreover, a recent study recognizes the emergence of a prometastatic state in human colon cancer cells upon imminent cell death, characterized by increased ER stress, cytokine release and stemness. The acquisition of the prometastatic state is found to be dependent on PERK–CHOP activation. Conversely, the inhibition of PERK is observed to enhance cell migration, suggesting that the resolution of ER stress is a prerequisite for metastasis.^264^ It is also noteworthy that the UPR has been demonstrated to influence the recognition of cancer cells by immune cells. The activation of IRE1α–XBP1s and PERK in melanoma cells has been demonstrated to suppress the expression of stress‐induced ligands for natural killer cells, including MHC class I polypeptide‐related sequence A and B (MICA and MICB) and B7 homolog 6 (B7H6).^265^ This leads to the inhibition of killing cancer cells mediated by natural killer cells.

## 5 THERAPEUTIC POTENTIAL

In light of the mounting evidence linking ER stress and UPR response involvement in the etiology of a plethora of human pathologies, there is a growing interest in the potential pharmacological modulation of its outputs to regulate cell fate during ER stress. Therefore, It is evident that UPR pathways represent a promising avenue for the development of novel therapeutic strategies, aiming to modulate ER stress and associated diseases^244^ (Table 4).

**TABLE 4:** Preclinical development and clinical trials on UPR modulators.

Compound/company	Type	Mechanism/target	Status	References
Sunitinib/Pfizer	IRE1 (RNase) activators	Type I kinase inhibitor	In clinical use	266
GSK2850163/GlaxoSmithKline	IRE1 inhibitors	Type III kinase inhibitor	Preclinical development	267
KIRA6 (compound 3 analogue)		Type II kinase inhibitor	Preclinical development	268, 269
KIRA7 (compound 3 analogue)		Type II kinase inhibitor	Preclinical development	159, 270
KIRA8 (AMG‐18)		Type II kinase inhibitor	Preclinical development	159
AD60		Type II kinase inhibitor	Preclinical development	271
CCT020312	PERK activators	Unclear	Preclinical development	272
MK‐28		Unclear	Preclinical development	273
ISRIB	eIF2B activators	Stabilizes eIF2B	Preclinical development	274
Guanabenz	PPP1R15 inhibitors	Putative PPP1R5A inhibitor	Phase I trial terminated (NCT02423083)	275

Abbreviations: DFG, Asp‐Phe‐Gly; type I kinase inhibitors bind in active‐state (DFG‐in) conformations; type II kinase inhibitors bind inactive‐state (DFG‐out) conformations; type III kinase inhibitors bind next to the ATP site; KIRA, kinase inhibiting RNase attenuator and inactive state.

### 5.1 Inducing adaptive UPR to reduce ER stress

In degenerative diseases such as neurodegeneration, a shift in the balance of the UPR toward cell survival rather than apoptosis may potentially alter the course of the disease. Nevertheless, the interrelated nature of the UPR may necessitate the simultaneous targeting of several components to achieve the most favorable outcomes. One potential strategy may be to extend the adaptive phase of the UPR, thereby increasing the likelihood of recovery. This may be accomplished by targeting transcription factors such as XBP1 and ATF6. An alternative approach is to inhibit key mediators of apoptosis, such as thioredoxin‐interacting protein (TXNIP) and CHOP. The targeting of potential timing regulators of the UPR, such as growth arrest and DNA damage‐inducible protein (GADD34) and DNAJ homolog subfamily C member 3 (DNAJC3), may prove to be a promising avenue of research. For example, compound salubrinal has been demonstrated to enhance cellular survival in the context of ER stress by inhibiting phosphatases that are involved in the dephosphorylation of eIF2α.^276^ Furthermore, small chemical chaperones such as TUDCA, which facilitate protein folding and exert a broad influence on stabilizing protein structures, have demonstrated encouraging protective effects in preclinical models of diabetes^277^, ^278^ and IBD.^190^ The presented examples demonstrate the evolving comprehension of the function of ER stress in disease and the prospective therapeutic advantages of targeting UPR components with small chemical chaperones to regulate cell fate under conditions of ER stress. In Europe, this chaperone has been approved for the treatment of cholelithiasis and cholestatic liver disease. Clinical trials have demonstrated the safety and efficacy of this treatment when administered to patients for a period between 1 and 6 months. It has been demonstrated to be efficacious in the treatment of a range of disorders, including primary biliary cirrhosis, HCV‐related chronic hepatitis, liver cirrhosis, and insulin resistance.^279^, ^280^ Currently, numerous pharmaceuticals approved by the US Food and Drug Administration (FDA) exert a portion of their therapeutic effects by restoring ER homeostasis. For example, macelignan has been demonstrated to enhance insulin sensitivity and improve lipid metabolic disorders by activating PPARα/γ and attenuating ER stress. These findings suggest that macelignan may have the potential as an antidiabetic agent for the treatment of type 2 diabetes.^281^ In addition, the glucagon‐like peptide‐1 receptor agonist exendin‐4 has been shown to alleviate ER stress in pancreatic islet β‐cells in vivo by upregulating the expression of ATF4. Specific pharmacological chaperones have been developed to facilitate the folding and trafficking of several proteins, including G protein‐coupled receptors, lysosomal enzymes and the Δ508Phe mutant cystic fibrosis transmembrane conductance regulator, which are misfolded as a result of gene mutations.^282^ It is also possible to alleviate ER stress in vivo through genetical therapy. For example, the delivery of an active form of XBP1 via AAV has been demonstrated to markedly diminish the buildup of mutant proteins in neurons in a mouse model of HD.

### 5.2 Induction of ER stress for cancer therapy

Continuous activation of the UPR response influences malignant progression, tumor development, and metastasis. Consequently, therapies aimed at disrupting ER stress and UPR signaling pathways can be utilized to directly kill tumors and promote concurrent antitumor immune responses.^283^ Such therapeutic modalities have the potential to induce a state of severe ER stress, ultimately leading to the demise of cancerous cells. The US FDA has approved the use of bortezomib, a proteasome inhibitor, as a cancer therapy, which can induce a unique type of ER stress characterized by an absence of eIFα phosphorylation, the accumulation of ubiquitylated proteins, and proteotoxicity.^284^ Some drugs target specific parts of the protein‐folding or UPR pathways, including HSP90, BiP, IRE1α, PERK, and GRP94, resulting in the death of cancer cells due to stress. Because there is a link among BiP expression, tumor stage, and drug resistance, anti‐BiP therapies are being tested in different cancers.^285^ As an alternative approach, the use of therapeutic agents that activate the PERK–ATF4–CHOP pathway has been shown to increase the apoptotic in cells with constitutive UPR activation.^286^ The UPR inhibition or PERK activation could enhance the efficacy of other therapies, offering a competitive advantage in combination therapies.^285^ Furthermore, small‐molecule KP1339, ruthenium‐based, has demonstrated efficacy in targeting BiP, with disease control observed in 26% of patients with advanced solid tumors in the Phase 1 clinical trial.^287^, ^288^ Another strategy for tumors is to induce lethal ER stress in malignant cells by activating the UPR signaling pathways. The HA15 compound, a BiP inhibitor, has been observed to induce cell death and autophagy tested in melanoma models ^289^ Further UPR‐activating compounds have been identified, including IXA4 (an IRE1α–XBP1s activator), MK‐28 (a PERK activator), and AA147 (an ATF6 activator),^290^ but their antitumor activity in vivo has yet to be fully defined. In addition, ER stress within the TME affects the function of cancer‐supporting stromal cells and inhibits tumor growth. Nevertheless, it is essential to exercise caution when developing therapies that target UPR components, because both pro‐ and antisurvival effects on cells have been observed by the activation of the UPR signaling pathway. In conclusion, it represents a highly promising approach to developing therapies targeting ER stress and UPR signaling pathways for the treatment of cancer.

## 6 CONCLUSION AND PERSPECTIVE

The ER is a very important organelle in maintaining cell homeostasis and its role in the cell survival and death signaling pathway is crucial. ER stress response is a self‐protective mechanism of the cell itself, a signaling response pathway system, and involves the regulation of the expression of various genes. A certain level of ER stress response can activate the expression of protective molecules such as ER chaperones, allowing cell to resist stress and maintain cell survival. However, too much or too prolonged ER stress can result in cell dysfunction, even cell death, and other pathological phenomena. ER stress has been implicated in the onset and development of many diseases. The selection of diseases in this review is quite comprehensive, covering different types of diseases, including CVDs, neurodegenerative diseases, metabolic diseases, autoimmune diseases, fibrotic diseases, and cancer, representing different disease categories and systems, each of which may be associated with ER stress.

Over the last few decades, our understanding of the role of ER stress in disease has progressed, but many important questions remain. It is generally accepted that there are two ways of targeting the UPR to treat different types of disease: one is to activate components in the compensatory pathway of the UPR to cope with stress; the other is to inactivate components in the proapoptotic pathway of the UPR. Although targeting the UPR represents a promising avenue for the treatment of various diseases, our current understanding is constrained by several limitations. For instance, as ER stress sensors, IRE1α and PERK initiate signaling pathways to maintain ER homeostasis and induce cell death. However, how these signaling pathways transition from cell survival to cell death has implications for the activation or inhibition of ER stress during treatment. It remains unclear to what extent the compensatory and proapoptotic pathways of the UPR contribute to the pathophysiology of various diseases. Furthermore, it is currently unknown whether activating or inactivating components of the UPR could ameliorate disease states. This lack of knowledge leaves a significant gap in the precise dose and timing of targeting reagents for application.

For different types of diseases, there should be more specific considerations for targeting ER stress or the UPR signaling pathways. For example, in CVDs, we need to explore how cardiomyocyte excitation‐contraction coupling and ER stress are involved in the process of calcium release during cardiomyocyte apoptosis. Although recent studies have shown that chemical ER chaperones can reduce ER stress in some diseases, the ER is a multifunctional organelle in a wide variety of normal and diseased cells, so how can drugs be delivered to target tissues or cells? Therefore, it is imperative to develop tissue‐ or cell‐specific drug delivery systems. Understanding the molecular mechanism of UPR activation and ER stress‐mediated apoptosis in different diseases will undoubtedly help us to understand diseases from a new perspective and provide new targets and therapeutic strategies for drug development for diagnosis and treatment of related diseases.

## AUTHOR CONTRIBUTIONS

Yingying Liu performed the literature search, drafted the manuscript, and critically revised it. Chunling Xu assisted in creating tables. Renjun Gu, Ruiqin Han, and Ziyun Li edited and revised the manuscript. Xianrong Xu supervised and revised the manuscript. All authors have read and approved the final manuscript.

## CONFLICT OF INTEREST STATEMENT

The authors declare no conflict of interest.

## ETHICS STATEMENT

Not applicable.

## Data Availability

Not applicable.
